# Characteristics, survival, and related factors of newly diagnosed colorectal cancer patients refusing cancer treatments under a universal health insurance program

**DOI:** 10.1186/1471-2407-14-446

**Published:** 2014-06-17

**Authors:** Chun-Yi Liu, William Tzu-Liang Chen, Pei-Tseng Kung, Chang-Fang Chiu, Yueh-Hsin Wang, Shwn-Huey Shieh, Wen-Chen Tsai

**Affiliations:** 1 Department of Health Services Administration, China Medical University, 91 Hsueh-Shih Road, Taichung 40402, Taiwan; 2 Department of Surgery, China Medical University Hospital, Taichung, Taiwan; 3 Department of Healthcare Administration, Asia University, Taichung, Taiwan; 4 Division of Hematology and Oncology, Department of Internal Medicine, China Medical University Hospital, Taichung, Taiwan; 5 Department of Education, China Medical University Hospital, Taichung, Taiwan; 6 Internal Medicine, College of Medicine, China Medical University, Taichung, Taiwan

**Keywords:** Colorectal cancer, Refusing treatment, Survival, Universal health insurance program

## Abstract

**Background:**

Colorectal cancer is the third most commonly diagnosed cancer worldwide. Few studies have addressed the causes and risks of treatment refusal in a universal health insurance setting.

**Methods:**

We examined the characteristics and survival associated with treatment refusal in patients with newly diagnosed colorectal cancer in Taiwan during 2004–2008. Treatment refusal was defined as not undergoing any cancer treatment within 4 months of confirmed cancer diagnosis. Patient data were extracted from four national databases. Factors associated with treatment refusal were identified through logistic regression using the generalized estimating equation method, and survival analysis was performed using the Cox proportional hazards model.

**Results:**

Of the 41,340 new colorectal cancer cases diagnosed, 3,612 patients (8.74%) refused treatment. Treatment refusal rate was higher in patients with less urbanized areas of residence, lower incomes, preexisting catastrophic illnesses, cancer stages of 0 and IV, and diagnoses at regional and district hospitals. Logistic regression analysis revealed that patients aged >75 years were the most likely to refuse treatment (OR, 1.87); patients with catastrophic illnesses (OR, 1.66) and stage IV cancer (OR, 1.43) had significantly higher refusal rates. The treatment refusers had 2.66 times the risk of death of those who received treatment. Factors associated with an increased risk of death in refusers included age ≥75 years, insured monthly salary ≥22,801 NTD, low-income household or aboriginal status, and advanced cancer stage (especially stage IV; HR, 11.33).

**Conclusion:**

Our results show a lower 5-year survival for colorectal patients who refused treatment than for those who underwent treatment within 4 months. An age of 75 years or older, low-income household status, advanced stages of cancer, especially stage IV, were associated with higher risks of death for those who refused treatment.

## Background

According to World Health Organization, cancer caused 7.6 million deaths (around 13% of all deaths) worldwide in 2008, and cancer-related deaths are expected to continue to rise. In 2008, approximately 70% of cancer deaths occurred in low-income and middle-income countries. Cancer could affect over 13 million people worldwide by the year 2030 [1]. In the United States, colorectal cancer is the third most common cancer in both men and women and is also the third leading cause of cancer-related deaths [2], with an age-adjusted annual incidence per 100,000 of 51.7 in men and 39.1 in women in 2006–2010, down from 57.2 and 42.5, respectively, in 2003–2007 [3]. In Taiwan, colorectal cancer is on the rise, and the age-adjusted annual incidence per 100,000 increased from 37.99 in men and 29.29 in women in 2003 to 54.39 in men and 36.84 in women in 2010 [4], rivaling the rates observed in the United States. According to Taiwan Cancer Registry’s vital statistics for new cancer cases in 2006–2010 (followed up until 2011), the five-year survival rates for colorectal cancer in men and women were 60.1% and 60.6%, respectively [5].

Most, but not all, patients diagnosed with colorectal cancer receive treatment. In the United States, proportion of colon cancer patients who received no treatment in 2012 was 1% for stages I and II, <1% for stage III, and 12% for stage IV; among rectal cancer patients, the proportions were 2% for stages I and II and 4% for stages III and IV [6]. Huchcroft and Snodgrass found that 7.5 out of 1000 cancer patients refused treatment [7]. A small study in older cancer patients observed a rate of 15.2% for partial or complete treatment refusal [8]. Various studies have found that 15.2% of patients refused recommended chemotherapy [9]; 8% of cancer patients did not undergo conventional cancer treatment [10]; 32% failed to complete cancer treatment [11]; and among breast cancer patients, 6% of those less than 65 years old and 22.2% of those 65 years or older did not receive standard treatment [12]. As most of the above studies were small-scale analyses, we were interested in conducting a nationwide study to examine treatment refusal among colorectal cancer patients in Taiwan.

A limited number of studies have examined the risks and possible causes of under treatment of cancer. One study conducted in Israel found that cancer patients who dropped out of chemotherapy, while not experiencing significantly different quality of life than those who refused the therapy, had a lower quality of life than patients who completed chemotherapy [13]. A systematic literature review concluded that chemotherapy has similar effectiveness and safety for elderly (aged ≥65 years) and nonelderly colon cancer patients, suggesting that elderly patients should be prescribed chemotherapy [14]. One study found that 27% of stage III colon cancer patients in Alberta, Canada, did not receive chemotherapy, and in 18% of these cases, the reason was the physicians’ not recommending the therapy, frequently because of the presence of one or more comorbidities [15]. Some terminal cancer patients’ refusal or avoidance of treatment was found to be related to a desire for death stemming from depression and feelings of hopelessness [16]. However, medical care professionals’ active surveillance and planning of future treatments were shown to positively influence patients’ desire to follow through with treatment [17].

Taiwan’s National Health Insurance program was launched in 1995 to ensure health insurance coverage for all people in Taiwan. Currently with a 99.9% coverage, this program has succeeded in making quality health care universal and affordable for patients [18]. The insurance premiums of low-income households are paid by the government, and copayments for the average person are also very low. Furthermore, copayments are waived for catastrophic illnesses and injuries (including cancer) in order to lower the barriers to health care access. Nevertheless, a small proportion of cancer patients in Taiwan choose not to undergo treatment for their cancer, and few data are available on the causes of this treatment refusal. In this study, we focused on colorectal cancer patients in Taiwan who refused treatment, and examined the patients’ characteristics, survival risks, and related factors.

## Methods

### Study population and data collection

During 2004–2008, a total of 52,324 new cases of colorectal cancer were diagnosed in Taiwan [19]. Of these, we included 41,340 cases classified as International Classification of Diseases for Oncology (ICD-O) codes C180–C218, which amounted to 79% of all colorectal cancer cases nationwide during that period. Treatment refusal was defined as not undergoing conventional cancer treatment (including surgery, radiation therapy, chemotherapy, radiation therapy with concurrent chemotherapy, and targeted therapy) more than 4 months of confirmed cancer diagnosis. This 4-month period was chosen because it is also the time frame of cancer treatment used by Taiwan’s National Cancer Registry. As reported by Tsai in 2013, the cumulative treatment rate for colorectal cancer was 87.71% at 1 month of diagnosis, 94.01% at 4 months, 94.35 at 5 months, and 95.48% at 12 months [20], indicating very little increase in the cumulative treatment rate from the fifth month, thus supporting our assumption of treatment refusal for newly diagnosed cancer patients who remained untreated after 4 months. Cancer patients receiving only palliative care were excluded from our analysis.

Cancer registration in Taiwan began in 1979. The patient data analyzed in this study were retrieved from four complete databases as follows. (i) The 2004–2008 Taiwan Cancer Registry Database (TCRD) of Taiwan’s Health Promotion Administration, Ministry of Health and Welfare provided data on sex, age at diagnosis, cancer stage, and treatment vs. no treatment. (ii) The National Health Insurance Research Database (NHIRD) of Taiwan’s National Health Insurance Administration provided data on sex, age at diagnosis, urbanization level of residence location, insured monthly salary, low-income household status, other catastrophic illnesses or injuries, comorbidity for calculating Charlson Comorbidity Index (CCI), level of diagnosing or treatment hospital, and ownership type of diagnosing or treatment hospital. (iii) Taiwan’s cause-of-death database profile: survival or not at the end of 2010 provided data on post-diagnosis survival in deceased patients. (iv) The Aboriginal Committee’s aboriginal status records of Taiwan’s Ministry of the Interior provided data on aboriginal status. Urbanization levels of residence locations were assigned according to Liu et al.’s classification system, which includes seven levels (from the most to the least urbanized): level 1, highly urbanized cities; level 2, moderately urbanized cities; level 3, developing cities; level 4, average towns; level 5, aging towns; level 6, agricultural towns; and level 7, remote villages [21].

### Statistical analysis

To analyze factors associated treatment refusal, we chose to test a set of patient and hospital characteristics, including the following variables: basic personal characteristics (sex, age at diagnosis, urbanization level of residence location, and aboriginal status), socioeconomic status (insured monthly salary and low-income household status), health status (preexistence of catastrophic illnesses or injuries other than cancer, and cancer stage), and characteristics of the diagnosing hospital (hospital level and hospital ownership type). First, we described the 2004–2008 patient set in terms of the proportion that received treatment and the proportion that refused treatment, and stratified the two groups by patient and hospital characteristics. Next, Student’s *t* test and the chi-square test were applied to determine whether the treated and untreated patients differed in patient or hospital characteristics. In addition, in order to adjust for the cluster effect, logistic regression was performed using the generalized estimating equation (GEE) method to identify those factors that correlated with treatment refusal.

To analyze effects on patient survival, we first computed the 1- through 5-year survival rates for the treated and untreated patients, and then examined by ANOVA whether post-diagnosis survival differed among deceased patients with different stages of cancer. The starting point of the survival analysis was a confirmed cancer diagnosis, and the endpoint was patient death or the last follow-up on survival status at the end of 2010, whichever was sooner. We also examined by the chi-square test whether survival differed among patients differing in basic personal characteristics, socioeconomic status, health status, severity of cancer, and hospital characteristics. Finally, survival analysis using the Cox proportional hazards model was performed, while controlling for the contributions of individual patient and hospital factors, in order to analyze the effect of treatment refusal on survival and the factors that influenced survival in treatment refusers. Statistical analysis was performed with SAS software, version 9.3.

The Statistics Center of Department of Health, Taiwan, helped us combine all data sets with personal identification number and then provided us the data sets including the necessary information for this study. All personal identification information has been deleted, and personal privacy was under protection from using these data. This study has been approved by the research ethics committee in China Medical University and Hospital (IRB No. CMUH102-REC3-076).

## Results

During 2004–2008, a total of 42,340 new cases of colorectal cancer were diagnosed, in 3,612 of which the patient refused treatment. The various patient and hospital characteristic for these cases are described in Table 1. In terms of patient baseline characteristics, the proportion of treatment-refusing (untreated) patients tended to increase with increasing age at diagnosis, whereas the proportion of treated patients was similar in all age groups, but was the lowest in the ≥75 years group. The treatment-refusing group was on average 4.6 years older than the treated group (69.52 ± 14.47 vs. 64.92 ± 13.58 years); patients residing in more urbanized areas had lower rates of treatment refusal, as did patients with higher monthly income levels (refusal rate dropping from 9.57% for insured dependents to 5.70% for the ≥22,801 NTD group); higher treatment-refusal rates were observed in patients with preexisting catastrophic illnesses or injuries (16.12% vs. 8.45% without such preexisting conditions) and in patients with stage 0 and stage IV cancer (8.15% and 12.98%). Among deceased patients who had refused treatment, those with stage III cancer survived the longest post-diagnosis (1.62 ± 1.31 years), while those with stage IV cancer survived for the shortest time (0.53 ± 0.77 years). Among deceased patients who had received treatment, post-diagnosis survival was the longest for stage I cancer (2.32 ± 1.64 years) and the shortest for stage IV cancer (1.23 ± 1.01 years). Treated patients of every cancer stage lived longer post-diagnosis than untreated patients of the same cancer stage. In terms of characteristics of the diagnosing hospital, patients diagnosed at medical centers—or the highest hospital level—had the lowest treatment refusal rate (6.22%). Sex, aboriginal status, and ownership type of the diagnosing hospital did not have statistically significant correlations with patients’ refusing or undergoing treatment (P > 0.05).

**Table 1:** Patient and hospital characteristics of colorectal cancer patients, by treatment choice (untreated vs. treated)

**Variable**	**No. of patients**	**Untreated**		**Treated**		**P value**
		**N**	**%**	**N**	**%**	
**Total colon cancer patients**	41,340	3,612	8.74	37,728	91.26	
**Sex**						0.893
Female	17,862	1,565	8.76	16,297	91.24	
Male	23,478	2,047	8.72	21,431	91.28	
**Age at diagnosis, years**						<0.001*
≤44	3,113	209	6.71	2,904	93.29	
45–54	6,298	426	6.76	5,872	93.24	
55–64	8,584	539	6.28	8,045	93.72	
65–74	11,219	843	7.51	10,376	92.49	
≥75	11,991	1,572	13.11	10,419	86.89	
**Age at diagnosis, years (M ± SD)**	41,340	69.52	14.47	64.92	13.58	<0.001*
**Urbanization level of residence location**						0.012*
Level 1 (highest)	12,201	1,015	8.32	11,186	91.68	
Level 2	11,812	1,031	8.73	10,781	91.27	
Level 3	6,211	529	8.52	5,682	91.48	
Level 4	6,133	544	8.87	5,589	91.13	
Level 5	1,230	115	9.35	1,115	90.65	
Level 6	1,876	207	11.03	1,669	88.97	
Level 7 (lowest)	1,738	148	8.52	1,590	91.48	
**Aboriginal status**						0.501
No	41,115	3,589	8.73	37,526	91.27	
Yes	225	23	10.22	202	89.78	
**Insured monthly salary, NTD**						<0.001
≤17,280	8,551	850	9.94	7,701	90.06	
Insured dependent	12,728	1,218	9.57	11,510	90.43	
17,281–22,800	13,153	1,134	8.62	12,019	91.38	
≥22,801	6,769	386	5.70	6,383	94.30	
**Low-income household status**						0.008*
No	30,766	2,508	8.15	28,258	91.85	
Yes	363	44	12.12	319	87.88	
**Other catastrophic illnesses or injuries**						<0.001*
No	39,814	3,366	8.45	36,448	91.55	
Yes	1,526	246	16.12	1,280	83.88	
**Cancer stage**						<0.001*
Stage 0	1,559	127	8.15	1,432	91.85	
Stage I	6,478	195	3.01	6,283	96.99	
Stage II	9,078	204	2.25	8,874	97.75	
Stage III	12,254	313	2.55	11,941	97.45	
Stage IV	9,131	1,185	12.98	7,946	87.02	
Other/Unknown	2,840	1,588	55.92	1,252	44.08	
**Post-diagnosis survival in deceased patients, years (M ± SD)**						<0.001*
Stage 0	1,559	1.57	1.81	1.97	1.47	
Stage I	6,478	1.61	1.46	2.32	1.64	
Stage II	9,078	1.19	1.28	2.08	1.51	
Stage III	12,254	1.62	1.31	2.00	1.39	
Stage IV	9,131	0.53	0.77	1.23	1.01	
**Level of diagnosing hospital**						<0.001*
Medical center	56,511	1,649	6.22	24,862	93.78	
Regional hospital	12,024	1,431	11.90	10,593	88.10	
District hospital	1,464	280	19.13	1,184	80.87	
**Ownership type of diagnosing hospital**						
Public	12,169	1,057	8.69	11,112	91.31	0.416
Private	28,325	2,389	8.43	25,936	91.57	

*P < 0.05.

To analyze patient and hospital factors related to treatment refusal, we performed logistic regression analysis using a generalized estimating equation (GEE) model (Table 2). We found that treatment refusal rate increased significantly with increasing patient age at diagnosis, with the highest rate being observed in patients aged ≥75 years (adjusted odds ratio [adj. OR], 1.87; 95% confidence interval [95% CI], 1.55–2.26). Treatment refusal rate also decreased significantly with increasing insured monthly salary (adj. OR, 0.79; 95% CI, 0.69–0.89) and increased significantly with preexisting catastrophic illnesses or injuries (adj. OR, 1.66; 95% CI, 1.37–2.02). In terms of cancer stage, treatment refusal rate was significantly higher in stage IV patients (adj. OR, 1.32; 95% CI, 1.03–1.69) and significantly lower in stage I through III patients (adj. OR, 0.38, 0.29, and 0.35) relative to stage 0 patients as the reference group. With regard to level of the diagnosing hospital, cancer diagnoses made at regional hospitals (adj. OR, 1.54; 95% CI, 1.09–2.17) and district hospitals (adj. OR, 1.82; 95% CI, 1.27–2.61) were significantly more likely to result in treatment refusal than those made at medical centers, the reference group. Patients with higher CCI scores were more likely to refuse treatment. Sex, aboriginal status, low-income household status, and hospital ownership type were not significantly correlated with treatment refusal (P > 0.05).

**Table 2:** GEE logistic regression analysis of correlations between patient and hospital characteristics and treatment refusal in colorectal cancer patients

**Variable**	**GEE analysis**^**a**^			
	**Adj. OR**	**95% CI**		**P value**
**Sex**				
Female (reference group)	-	-	-	-
Male	1.06	0.98	1.14	0.172
**Age at diagnosis, years**				
≤44 (reference group)	-	-	-	-
45–54	1.13	0.97	1.31	0.112
55–64	1.14	1.01	1.29	0.028*
65–74	1.24	1.06	1.45	0.009*
≥75	1.87	1.55	2.26	<0.001*
**Urbanization level of residence location**				
Level 1 (highest; reference group)	-	-	-	-
Level 2	0.97	0.86	1.08	0.555
Level 3	0.90	0.78	1.04	0.144
Level 4	0.89	0.76	1.05	0.172
Level 5	1.01	0.82	1.24	0.961
Level 6	1.01	0.82	1.23	0.961
Level 7 (lowest)	0.96	0.76	1.22	0.761
**Aboriginal status**				
No (reference group)	-	-	-	-
Yes	0.80	0.51	1.24	0.317
**Insured monthly salary, NTD**				
≤17,280 (reference group)	-	-	-	-
Insured dependent	0.78	0.65	0.94	0.007*
17,281–22,800	0.90	0.80	1.00	0.048*
≥22,801	0.79	0.69	0.89	<0.001*
**Low-income household status**				
No (reference group)	-	-	-	-
Yes	1.33	0.98	1.80	0.064
**Other catastrophic illnesses or injuries**				
No (reference group)	-	-	-	-
Yes	1.66	1.37	2.02	<0.001*
**Cancer stage**				
Stage 0 (reference group)	-	-	-	-
Stage I	0.38	0.29	0.48	<0.001*
Stage II	0.29	0.22	0.39	<0.001*
Stage III	0.35	0.27	0.47	<0.001*
Stage IV	1.32	1.03	1.69	0.029*
**Charlson Comorbidity Index (CCI)**				
≤3 (reference group)	-	-	-	-
4–6	1.29	1.07	1.56	0.009*
≥7	1.28	1.05	1.56	0.013*
**Level of diagnosing hospital**				
Medical center (reference group)	-	-	-	-
Regional hospital	1.54	1.09	2.17	0.013*
District hospital	1.82	1.27	2.61	0.001*
**Ownership type of diagnosing hospital**				
Public (reference group)	-	-	-	-
Private	0.86	0.63	1.17	0.330

^a^event: treatment refusal *P < 0.05.

**Figure 1:**
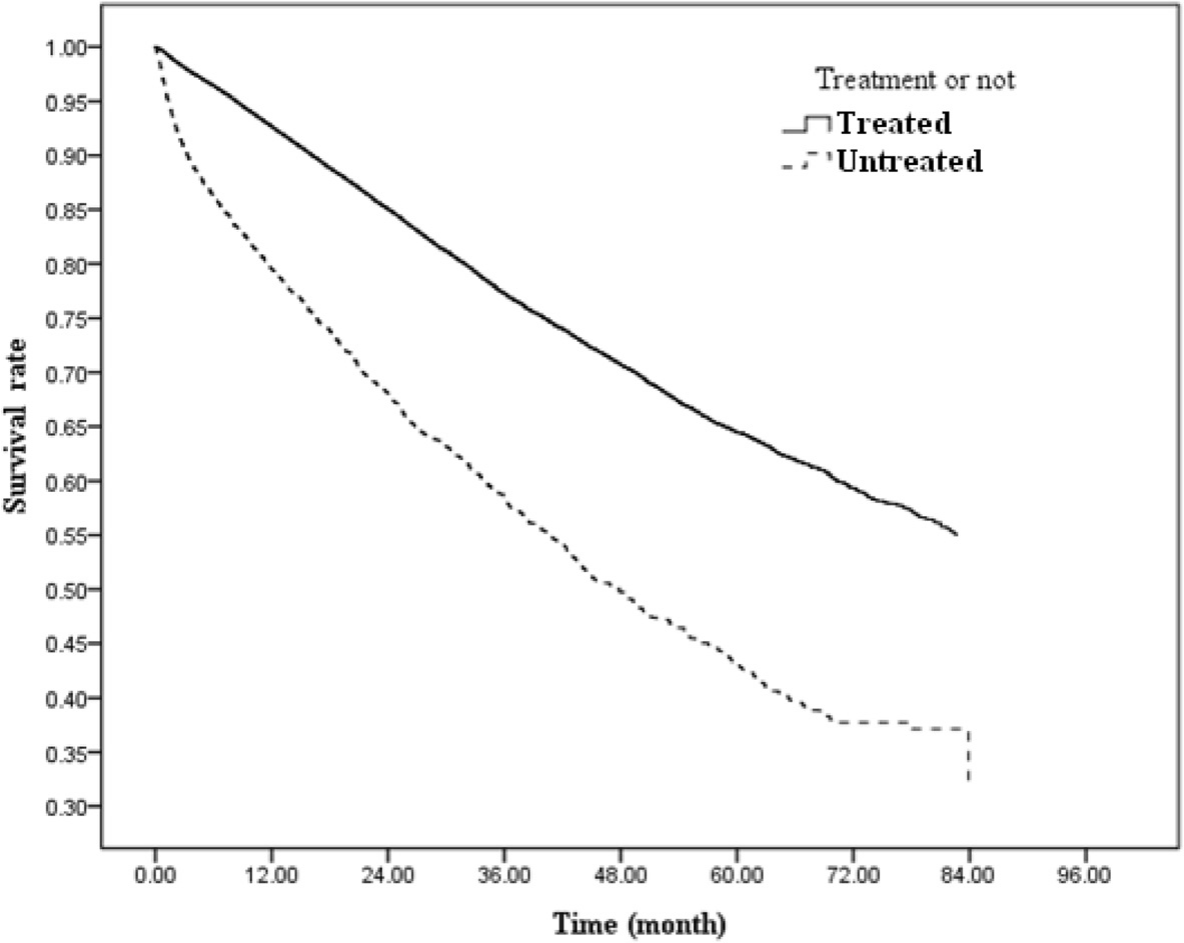
One- through five-year survival rates in treated and untreated colorectal cancer patients.

Next, we analyzed whether treatment refusal had an effect on the survival of colorectal cancer patients (Table 3). After other variables’ effects were adjusted for, we found that patients who refused treatment had 2.66 times the risk of death as those who underwent treatment (95% CI, 2.49–2.84). The risk of death was lower in male patients than in female patients (adj. HR, 0.92; 95% CI, 0.88–0.96) and was significantly higher in patients residing in the least urbanized (level 7) areas (adj. HR, 1.17; 95% CI, 1.07–1.28). The risk of death also increased significantly with increasingly advanced stage of cancer diagnosed, with stage IV patients being the most likely to die (adj. HR, 19.38; 95% CI, 15.96–23.54). When we analyzed the 1- through 5-year survival rates of treated and untreated patients (Figure 1), we found that these rates were significantly lower in treatment refusers than in treated patients.

**Table 3:** Effect of treatment refusal on survival in colorectal cancer patients

**Variable**	**Adj. HR**^**a**^	**95% CI**		**P value**
**Treatment**				
Yes (reference group)	-	-	-	-
No	2.66	2.49	2.84	<0.001*
**Sex**				
Female (reference group)	-	-	-	-
Male	0.92	0.88	0.96	<0.001*
**Age at diagnosis, years**				
≤44( (reference group)	-	-	-	-
45–54	0.86	0.80	0.93	<0.001*
55–64	0.89	0.82	0.96	0.002*
65–74	1.04	0.96	1.12	0.366
≥75	1.75	1.63	1.88	<0.001*
**Urbanization level of residence location**				
Level 1 (highest; reference group)	-	-	-	-
Level 2	0.97	0.92	1.02	0.233
Level 3	1.06	1.00	1.13	0.052
Level 4	1.05	0.99	1.12	0.112
Level 5	1.05	0.95	1.17	0.333
Level 6	1.05	0.96	1.14	0.306
Level 7 (lowest)	1.17	1.07	1.28	0.001*
**Aboriginal status**				
No (reference group)	-	-	-	-
Yes	0.89	0.71	1.12	0.328
**Insured monthly salary, NTD**				
≤17,280 (reference group)	-	-	-	-
Insured dependent	0.97	0.90	1.05	0.455
17,281–22,800	1.00	0.95	1.05	0.908
≥22,801	0.80	0.75	0.85	<0.001*
**Low-income household status**				
No (reference group)	-	-	-	-
Yes	1.53	1.32	1.79	<0.001*
**Other catastrophic illnesses or injuries**				
No (reference group)	-	-	-	-
Yes	1.83	1.68	2.00	<0.001*
**Cancer stage**				
Stage 0 (reference group)	-	-	-	-
Stage I	1.59	1.30	1.95	<0.001*
Stage II	2.63	2.16	3.21	<0.001*
Stage III	3.84	3.16	4.68	<0.001*
Stage IV	19.38	15.96	23.54	<0.001*

^a^event: death; the model has controlled for level of diagnosing hospital and ownership type of diagnosing hospital.

*P < 0.05.

Finally, we analyzed characteristics of treatment-refusing patients that might have affected these patients’ survival, and the results are presented in Table 4. Among treatment refusers, those aged ≥75 years had a significantly higher risk of death than those in other age groups (adj. HR, 1.59; 95% CI, 1.20–2.09); those who were aboriginals had a significantly lower risk of death (adj. HR, 0.33; 95% CI, 0.13–0.81), as did those who had the highest (≥22,801 NTD) insured monthly salary (adj. HR, 0.74; 95% CI, 0.60–0.92); belonging to a low-income household significantly raised the risk of death (adj. HR, 1.71; 95% CI, 1.07–2.72). In addition, higher CCI scores were associated with significantly increased risks of death, especially in patients with a CCI ≥7 (adj. HR, 15.96; 95% CI, 14.43–17.65). The risk of death in treatment refusers also increased significantly with increasingly advanced stage of cancer, with stage IV patients having the highest risk (adj. HR, 11.33; 95% CI, 7.55–17.00). Sex, urbanization level of residence location, and preexisting catastrophic illnesses or injuries were not significantly correlated with survival in treatment refusers (P > 0.05).

**Table 4:** Correlations between patient factors and survival in treatment-refusing colorectal cancer patients

**Variable**	**Adj. HR**^**a**^	**95% CI**		**P value**
**Sex**				
Female (reference group)	-	-	-	-
Male	1.03	0.91	1.17	0.650
**Age at diagnosis, years**				
≤44 (reference group)	-	-	-	-
45–54	0.79	0.59	1.07	0.132
55–64	0.93	0.69	1.25	0.630
65–74	0.96	0.72	1.28	0.769
≥75	1.59	1.20	2.09	0.001*
**Urbanization level of residence location**				
Level 1 (highest; reference group)	-	-	-	-
Level 2	0.89	0.75	1.05	0.158
Level 3	1.06	0.87	1.30	0.578
Level 4	1.04	0.85	1.28	0.692
Level 5	0.89	0.63	1.26	0.510
Level 6	0.90	0.68	1.18	0.433
Level 7 (lowest)	1.27	0.95	1.69	0.115
**Aboriginal status**				
No (reference group)	-	-	-	-
Yes	0.33	0.13	0.81	0.015*
**Insured monthly salary, NTD**				
≤17,280 (reference group)	-	-	-	-
Insured dependent	0.98	0.75	1.28	0.889
17,281–22,800	0.92	0.79	1.08	0.330
≥22,801	0.74	0.60	0.92	0.007*
**Low-income household status**				
No (reference group)	-	-	-	-
Yes	1.71	1.07	2.72	0.024*
**Other catastrophic illnesses or injuries**				
No (reference group)	-	-	-	-
Yes	1.26	0.98	1.60	0.070
**Charlson Comorbidity Index (CCI)**				
≤3 (reference group)	-	-	-	-
4–6	7.31	6.54	8.16	<0.001*
≥7	15.96	14.43	17.65	<0.001*
**Cancer stage**				
Stage 0 (reference group)	-	-	-	-
Stage I	1.48	0.92	2.38	0.110
Stage II	2.99	1.91	4.67	<0.001*
Stage III	3.58	2.35	5.46	<0.001*
Stage IV	11.33	7.55	17.00	<0.001*

^a^event: death; the model has controlled for level of diagnosing hospital and ownership type of diagnosing hospital.

*P < 0.05.

## Discussion

In this study, we examined patient- and hospital-related factors and survival risks associated with treatment refusal in newly diagnosed colorectal cancer patients in Taiwan during 2004–2008.

One study showed that cancer patients who were single, divorced, or widowed or who were female were more likely to refuse cancer treatment; the authors speculated that male cancer patients were more likely to undergo treatment because men are typically the providers of financial support for their families [22]. In contrast, another study that examined early-stage (stage I and stage II) lung cancer patients reported a higher proportion of men than women (66.7% vs. 33.3%) among the treatment refusers [23]. Our present study revealed no significant difference in gender distribution between treated and treatment-refusing colorectal cancer patients. The discrepancies between our finding and those of Kleffens et al. and Chadha et al. could have arisen from differences in the health insurance systems in the patients’ countries. Due to the national health insurance program that provides universal coverage in Taiwan, health care is highly accessible to all Taiwanese, thus minimizing the effect of economic factors on patients’ willingness to seek medical treatment.

The rate of treatment refusal has been shown to increase with patient age [23-25]. We found the mean age of colorectal cancer patients who refused treatment to be 69.52 years, or 4.6 more years than that of patients who underwent treatment; logistic regression analysis using the GEE method showed that the likelihood of treatment refusal increased significantly with increasing age (adj. OR for the ≥75 years age group, 1.87; 95% CI, 1.55–2.26). These findings are consistent with those of the three previous studies [23-25].

Huchcroft and Snodgrass’s study on treatment-refusing cancer patients in Canada during 1975–1988 showed that patients living in remote areas refused treatment at a higher rate than those living in urban areas because of lower access to medical care and lower availability of advanced medical technologies [7]. This finding differs from our finding of a lack of a statistically significant relationship between treatment refusal and urbanization level of residence location. We speculate that, owing to the National Health Insurance program, financial and access barriers to medical care are lower in Taiwan than in many Western countries, thus making access to care less of a factor in the treatment status of the patients in our study sample. With respect to socioeconomic characteristics, our study showed that the rate of treatment refusal decreased with increasing level of patient insured monthly salary, and the risk of death was significantly lower in the highest insured monthly salary group (≥22801 NTD). This result is in agreement with that of Yim et al.’s 2010 study, and indicates that the cost of medical treatment is one of the barriers to cancer patients’ compliance with prescribed treatments [26].

Previous studies have shown that multiple cancers, advanced-stage cancer, and worsening of disease are all factors that contribute to patients’ refusal of or dropout from treatment [7,25]. In our study, an advanced cancer stage and preexisting catastrophic illnesses or injuries were both significantly associated with higher rates of treatment refusal. Besides the above characteristics, other factors that influence patients’ decision to refuse or drop out of treatment have been shown to include health status, access to information on their condition, attitude toward their condition, interactive relationships with medical staff, and encouragement from medical staff [27]. In their 2012 study on breast cancer patients who opted for alternative therapies in place of conventional treatment, Citrin et al. showed that the patients’ perception of unsympathetic physician attitudes (“uncaring, insensitive, and unnecessarily harsh”), fear of side effects, and belief in the efficacy of alternative therapies (such as consumption of raw fruits and vegetables and nutritional supplements), were key factors in these patients’ refusal of conventional medical treatment [28]. In the present study, our GEE-based regression modeling also uncovered the level of the diagnosing hospital to be a factor in treatment refusal, as colorectal cancer patients diagnosed at regional and district hospitals were significantly less likely to undergo treatment. Therefore, we suggest that physicians at lower-level hospitals refer their patients to medical centers for second opinions and encourage the patients to actively seek treatment. One noteworthy finding from our study is that stage 0 patients actually refused treatment at a higher rate than stage 1 through III patients; this may mean that health care staffs need to follow up on stage 0 patients more closely to ensure their receiving proper treatment.

Kowalski and Carvalho’ 2000 study found that approximately 50% of untreated head and neck cancer patients die within 4 months of diagnosis [25]; Chadha et al. determined the mean survival for untreated early-stage lung cancer to be 11.9 months [23]. In the present study, we found that untreated colorectal cancer patients had lower 1- through 5- year survival rates and (for deceased patients) shorter mean survival than their treated counterparts. We also observed a higher risk of death in colorectal cancer patients who were older or had higher incomes, consistent with a previous study’s finding that oral cancer patients who were younger and more affluent had higher survival rates [29]. The 2010 study by Yim et al. showed that survival prospects were poorer for low-income cancer patients than for their higher-income counterparts [26]. Kevin et al. also reported in their 2011 study that low-income colon cancer patients in San Francisco, United States, had lower survival rates than their high-income counterparts, but income level had no such effect on cancer patient survival in Toronto, Canada, where a publicly funded health care system provides universal coverage [30]. All the evidence above indicates that economic or income inequality is an important factor affecting cancer patient survival.

Sant et al. analyzed data for 4,478 patients diagnosed with breast cancer in 1990–1992 and found that stage IV patients had 3.38 times the risk of death of stage I patients [31]; Ma et al.’s study on lung cancer patients found cancer stage to be the main factor affecting patient survival [32]. Consistent with these results, our study found that advanced stages of colorectal cancer were associated with higher risks of death, especially stage IV (adj. HR, 19.38). Another study showed that stage III colon cancer patients who received chemotherapy had significantly higher overall survival than those who refused it [33]. Similarly, a study from the Netherlands found that stage III colon cancer patients treated with chemotherapy during 1989–2006 had significantly lower risks of death than those without chemotherapy [34]. Yet another study showed that among cancer patients indicated for radiation therapy, those who refused it significantly differed from those who complied with it in terms of several variables (age, race, marital status, and tumor location) and had a median survival 75 months shorter than those who complied [35]. In our present study, treatment-refusing cancer patients had 2.66 times the risk of death of their treated counterparts, with the risk being significantly higher for stage IV patients (adj. HR, 11.33). This finding is consistent with those reported in the literature, and suggests that cancer patients should comply with treatments recommended by their physicians for better clinical outcomes.

Indigenous Australians in Queensland have a lower overall incidence of cancer, but a higher overall cancer mortality, than the total population [36]. In a study involving patients in the United States Military Health System, Andaya et al. found that among colon cancer patients younger than 50, non-Hispanic Blacks experienced significantly lower overall survival than non-Hispanic Whites [37]. In our study with colorectal cancer patients in Taiwan, although aboriginals had a somewhat higher proportion of treatment refusers than non-aboriginals (10.22% vs. 8.45%), aboriginal status was not correlated with either treatment refusal or survival. It is possible that our result differs from those of other studies because Taiwan’s aboriginals experience few or no barriers to health care access under the National Health Insurance system.

Taiwan implements a universal health care system and mandates the participation of its entire population in order to remove barriers to people’s utilization of medical services. Taiwan’s health administrations also target remote areas with a mobile health policy to promote the fairness and universality of health care. Nevertheless, our study showed that in this environment of low-barrier, high-access medical care, some cancer patients still refused treatment; more studies are needed to further examine the reasons behind this phenomenon.

This research was a database analysis focused on the personal characteristics, socioeconomic status, and partial state of health of treatment-refusing cancer patients. Due to the use of secondary databases, which did not collect information on such variables as patient health behaviors, health belief and health awareness, we were not able to further ascertain patients’ reasons for refusing treatment with respect to these other variables.

## Conclusion

Since its inception in 1995, Taiwan’s National Health Insurance program has achieved 99.9% coverage and succeeded in making quality health care universal and affordable for patients. However, among the 41,340 new colorectal cancer cases diagnosed, 8.74% refused treatment within 4 months. Our results show a lower 5-year survival and a 2.66-fold increased risk of death for colorectal patients who refused treatment than for those who underwent treatment within 4 months. Therefore, we urge newly diagnosed cancer patients to actively seek treatment in order to enhance their chances of survival.

We identified several important factors that correlated with treatment refusal, including age at diagnosis, urbanization level of residence location, insured monthly salary, low-income household status, preexisting catastrophic illnesses or injuries, cancer stage, and level of diagnosing hospital. We also found that an age of 75 years or older, low-income household status, advanced stages of cancer, especially stage IV, were associated with higher risks of death for those who refused treatment.

## Abbreviations

ICD-O: International classification of diseases for oncology; TCRD: Taiwan cancer registry database; NHIRD: National Health Insurance Research Database.

## Competing interests

The authors declare that they have no competing interests.

## Authors’ contributions

Conception and design was done by WTC, CYL, PTK, YHW, collection and assembly of data was done by WTC, CFC, YHW, SHS., data analysis and interpretation was done by WTC, CYL, PTK, YHW, manuscript writing was done by CYL, WTC, YHW, final approval of manuscript was done by all authors.

## Pre-publication history

The pre-publication history for this paper can be accessed here:

http://www.biomedcentral.com/1471-2407/14/446/prepub
